# SK Channels Modulation Accelerates Equilibrium Recovery in Unilateral Vestibular Neurectomized Rats

**DOI:** 10.3390/ph14121226

**Published:** 2021-11-26

**Authors:** Brahim Tighilet, Audrey Bourdet, David Péricat, Elise Timon-David, Guillaume Rastoldo, Christian Chabbert

**Affiliations:** 1 Aix-Marseille Université-CNRS, Laboratoire de Neurosciences Cognitives, LNC UMR 7291, Centre Saint Charles, Case C, 3 Place Victor Hugo, CEDEX 03, 13331 Marseille, France; audrey.BOURDET@univ-amu.fr (A.B.); david.pericat@univ-amu.fr (D.P.); el.timon-david@laposte.net (E.T.-D.); guillaume.rastoldo@univ-amu.fr (G.R.); christian.chabbert@univ-amu.fr (C.C.); 2 GDR Physiopathologie Vestibulaire-Unité GDR2074 CNRS, 13331 Marseille, France

**Keywords:** SK channels, apamin, vertigo, rat model, vestibular compensation, vestibular function recovery, unilateral vestibular neurectomy

## Abstract

We have previously reported in a feline model of acute peripheral vestibulopathy (APV) that the sudden, unilateral, and irreversible loss of vestibular inputs induces selective overexpression of small conductance calcium-activated potassium (SK) channels in the brain stem vestibular nuclei. Pharmacological blockade of these ion channels by the selective antagonist apamin significantly alleviated the evoked vestibular syndrome and accelerated vestibular compensation. In this follow-up study, we aimed at testing, using a behavioral approach, whether the antivertigo (AV) effect resulting from the antagonization of SK channels was species-dependent or whether it could be reproduced in a rodent APV model, whether other SK channel antagonists reproduced similar functional effects on the vestibular syndrome expression, and whether administration of SK agonist could also alter the vestibular syndrome. We also compared the AV effects of apamin and acetyl-DL-leucine, a reference AV compound used in human clinic. We demonstrate that the AV effect of apamin is also found in a rodent model of APV. Other SK antagonists also produce a trend of AV effect when administrated during the acute phase of the vertigo syndrome. Conversely, the vertigo syndrome is worsened upon administration of SK channel agonist. It is noteworthy that the AV effect of apamin is superior to that of acetyl-DL-leucine. Taken together, these data reinforce SK channels as a pharmacological target for modulating the manifestation of the vertigo syndrome during APV.

## 1. Introduction

Apamin is a globular peptide neurotoxin of 18 amino acids present in the apitoxin-bee venom [1]. It is a polypeptide with the following amino acid sequence: H-Cys-Asn-Cys-Lys-Ala-Pro-Glu-Thr-Ala-Leu-Cys-Ala-Arg-Arg-Cys-Gln-Gln-His-NH2. The two disulfuric bridges between Cys1-Cys11 and Cys3-Cys15 together with the seven hydrogen bonds make apamin a highly stable molecule. Dry bee venom is composed of 2–3% apamin [2]. Apamin is one of the main peptide blockers of small conductance calcium-activated potassium channels (SK channels) [3,4]. Electrophysiology experiments evaluating its ability to block SK currents revealed a differential affinity of apamin for the three types of SK channels: SK1 (also known as KCa2.1, encoded by the KCNN1 gene), which has the least affinity (EC50 0.7–12 nM), SK2 (referred to as KCa2.2, encoded by the KCNN2 gene), which has the highest affinity (EC50 27–140 pM), and SK3 (identified as KCa2.3, encoded by the KCNN3 gene), which has intermediate affinity (EC50 0.6–4 nM) [5,6]. Interestingly, rat SK1 channels are unresponsive to apamin, unlike those in humans [7]. Apamin readily crosses the blood-brain barrier, and iodo-apamin studies reveal a map of binding sites similar to the distribution of SK2 and SK3 in rodent brain [8,9]. In the mammal vestibular nuclei, a high density of the SK2 subunit was observed, followed by medium and low distributions for the SK1 and SK3 subunits, respectively [9]. We also note an approximately similar distribution pattern in structures connected to the vestibular nuclei (cerebellum and prepositus hypoglossal nucleus) which also contribute to the stabilization of gaze and posture.

SK channels are members of the voltage-insensitive calcium-activated potassium channel family. Upon elevation of the cytosolic calcium concentration, the channels open, allowing K^+^ ions to leave the cell as a function of the difference between the depolarized cell and K^+^ equilibrium potentials. Consequently, their activation leads to the cell repolarization (for a review, [10]). More specifically, SK channels are thought to regulate neuronal excitability by contributing to the slow component of synaptic after hyperpolarization (AHP) [11,12], thereby governing the firing frequency. Activation or upregulation of SK channels is expected to reduce the firing frequency of repetitive action potentials. With regard to these specific gating properties, we may speculate on the expected functional consequences of the upregulation of SK channels in the vestibular nuclei (VN) depending on the cell type (excitatory or inhibitory neurons) and the side considered (intact versus deafferented) [13].

In a previous study, we demonstrated in a unilateral vestibular neurectomy (UVN) cat model, that administration of apamin during the acute phase of the vestibular syndrome significantly reduced both the posturo-locomotor and vestibulo-ocular deficits. This was illustrated by the reduction of both the horizontal spontaneous nystagmus and the static and dynamic balance unsteadiness [13].

To study more thoroughly the antivertigo effect of apamin, we investigated in this follow-up study whether this effect could be reproduced in a rodent model of acute peripheral vestibulopathy, by other SK channel antagonists, and whether SK channel agonists may produce a comparable or opposite antivertigo effect. We also compared the antivertigo effect of apamin relative to that of the standard antivertigo drug acetyl D-L leucine (Tanganil^®^). With this aim, we performed UVN in adult rats following the same surgical approach previously detailed [14], and used suitable behavioral testing to assess the progression of the vestibular syndrome following the vestibular injury.

## 2. Results

### 2.1. Dose-Response Effect of Apamin

**Figure 1:**
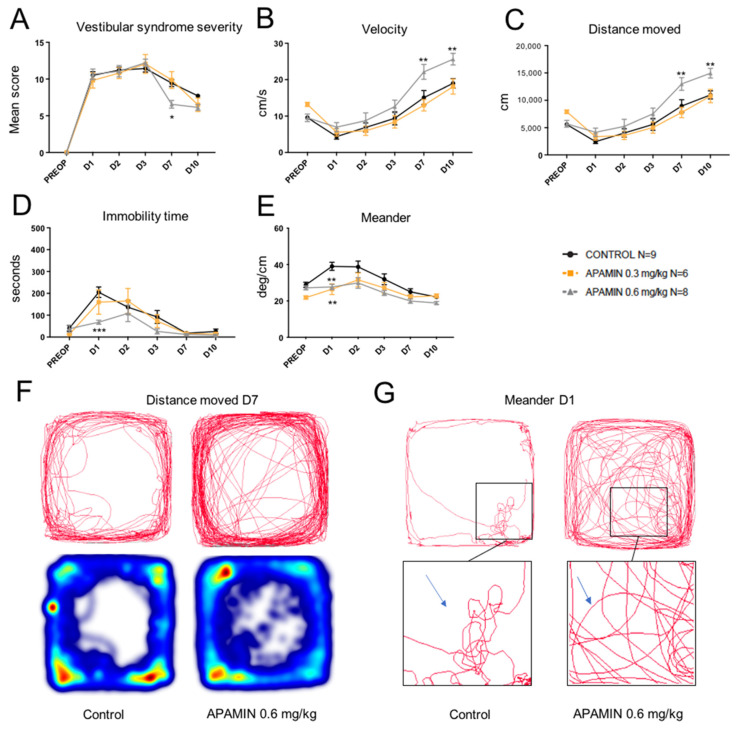
Effect of apamin treatment (0.3 and 0.6 mg/kg) on vestibular syndrome time course in UVN rats. (**A**), Subjective assessment of the vestibular syndrome severity over time (mean score established according to the rating scale detailed in Methods). (**B**), Measurement of the animal’s displacement speed (velocity, in cm/s) at the various time points considered. (**C**), Assessment of the total distance moved in the open field (in cm) during the recording session. (**D**), Immobility time (in s) of the animals during the videotracking recording phase. (**E**), Locomotor pattern of the animal (meander; °/cm). (**F**), Actimetry graphs emphasizing the difference in distance moved at D7 between control and apamin 0.6 mg/kg groups. Taken from C. (**G**), Movement tracing graphs emphasizing the difference in meander at D1 between control and apamin 0.6 mg/kg groups. Taken from E. Statistically significant differences (* *p* < 0.05; ** *p* < 0.01; *** *p* < 0.001) compared to the control group, two-way ANOVA.

**Figure 2:**
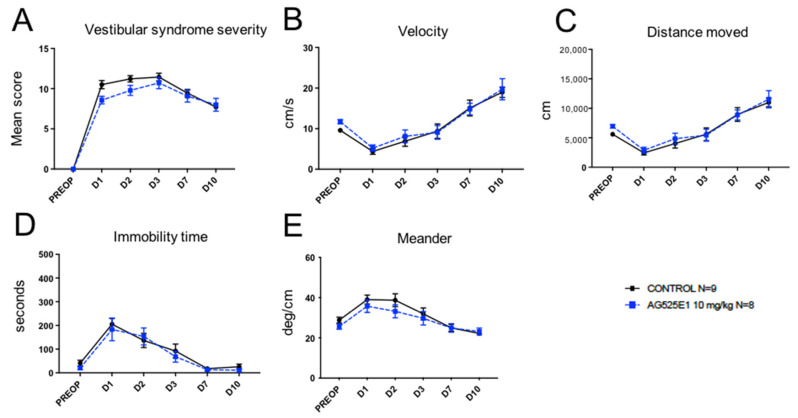
Effect of treatment with AG525E1 (10 mg/kg) on the severity of vestibular syndrome. (**A**), Subjective assessment of the vestibular syndrome severity over time. (**B**), Measurement of the animal’s velocity (in cm/s) at the various time points considered. (**C**), Assessment of the total distance traveled in the open field (in cm) during the recording session. (**D**), Immobility time (in s) of the animals during the videotracking recording phase. (**E**), Locomotor pattern of the animal (meander; °/cm). No statistically significant difference was observed at any time between AG525E1 and control rats.

When administered at 0.3 mg/kg, the apamin group did not display statistically significant differences vs. control group in most posturo-locomotor parameters considered, except for the meander which corresponds to the locomotor pattern (Figure 1). When administered at 0.6 mg/kg, apamin induced statistically significant benefit vs. control, which varied according to the parameter considered. The subjective evaluation of the vestibular syndrome severity over time revealed a significant reduction (F(6, 42) = 2.518; *p* = 0.0147) at D7 after the vestibular lesion, while it did not significantly vary vs. control at other time points (Figure 1A). The automated evaluation of posturo-locomotor parameters revealed a significant increase in the displacement velocity at D7 (F(6, 42) = 4.011; *p* = 0.0024) and D10 (F(6, 42) = 4.011; *p* = 0.0047; Figure 1B), and the total distance moved during the tests at D7 (F(6, 42) = 5.135; *p* = 0.0025) and D10 (F(6, 42) = 5.135; *p* = 0.0032; Figure 2C) in rats treated with apamin 0.6 mg/kg. Statistically, very significant reduction in immobility time (F(6, 42) = 5.602; *p* = 0.0009; Figure 1D) was observed at D1. Conversely to other posturo-locomotor testing, apamin administered at both 0.3 and 0.6 mg/kg displayed very significant reduction of altered locomotor pattern (meander) at D1 (F(6, 42) = 4.254; *p* = 0.0017 and *p* = 0.0085, respectively; Figure 1E). These observations indicated that acute ip administration of apamin at 0.6 mg/kg over the first days after UVN in adult rats induced a significant reduction in various behavioral biomarkers characteristic of vestibular disorders in rodents.

### 2.2. Effect of Other SK Channel Modulators

Administration to UVN rats of the bis-tetrahydroisoquinoline derivative (AG525E1) at 10 mg/kg, another SK channel antagonist, induced a trend to reduce the different studied parameters of the vestibular syndrome over the first two days following the unilateral vestibular lesion (Figure 2A–E), without reaching statistical difference versus control in our sample.

**Figure 3:**
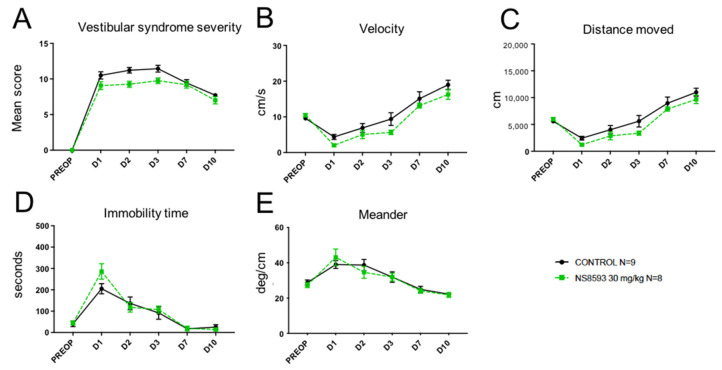
Effect of treatment with NS8593 (30 mg/kg) on the severity of vestibular syndrome. (**A**), Subjective assessment of the vestibular syndrome severity over time. (**B**), Measurement of the animal’s displacement speed (velocity; in cm/s) at the various time points considered. (**C**), Assessment of the total distance traveled in the open field (in cm) during the recording session. (**D**), Immobility time (in s) of the animals during the videotracking recording phase. (**E**), locomotor pattern of the animal (meander; °/cm). No statistically significant difference was observed at any time between AG525E1 and control rats.

Administration to UVN rats of NS8593 at 30 mg/kg, a negative modulator of SK2 channels, produced a trend to reduce the intensity of the vestibular syndrome (Figure 3A), immobility time (Figure 3D), and altered locomotion pattern (Figure 3E) over the first two days following the unilateral vestibular lesion, however without reaching statistical difference versus control in our sample. It evoked a similar tendency to reduce both the velocity (Figure 3B) and distance moved (Figure 3C) over all time points considered.

**Figure 4:**
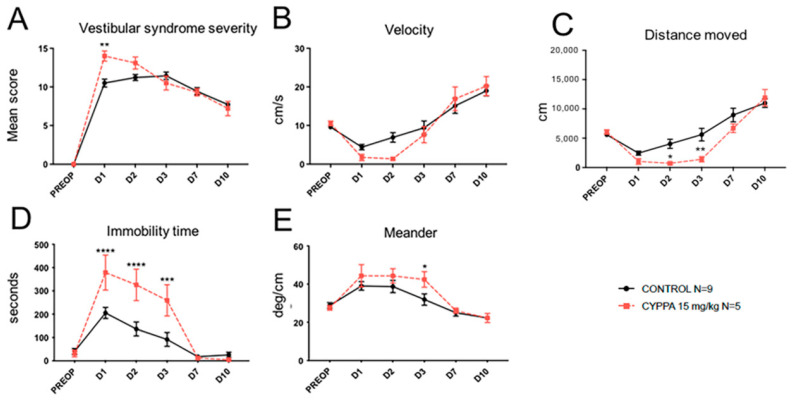
Effects of treatment with CyPPA (15 mg/kg) on the severity of vestibular syndrome. (**A**), Subjective assessment of the vestibular syndrome severity over time. (**B**), Measurement of the animal’s displacement speed (velocity; in cm/s) at the various time points considered. (**C**), Assessment of the total distance traveled in the open field (in cm) during the recording session. (**D**), Immobility time (in s) of the animals during the videotracking recording phase. (**E**), locomotor pattern of the animal (meander; °/cm). Statistically significant differences (* *p* < 0.05; ** *p* < 0.01; *** *p* < 0.001; **** *p* < 0.0001) compared to the control group, two-way ANOVA.

Administration to UVN rats of cyclohexyl-[2-(3,5-dimethyl-pyrazol-1-yl)-6-methyl-pyrimidin-4-yl]-amine (CyPPA), an SK channel agonist (Figure 4), induced significant (F(6, 42) = 2.518; *p* = 0.0055) increase in the severity of vestibular syndrome at D1 (Figure 4A) and a tendency to reduce the animal’s displacement velocity between D1 and D3, with a significant difference vs. control at D2 (F(6, 42) = 4.011; *p* = 0.049; Figure 4B). A significant decrease in the distance moved at D2 (F(6, 42) = 5.135; *p* = 0.0435) and D3 (F(6, 42) = 5.135; *p* = 0.0051) (Figure 4C), and a very significant increase in immobility time during the first three days of vestibular syndrome (F(6, 42) = 5.602; *p* = 0.0001 at D1, *p* < 0.0001 at D2 and *p* = 0.0002 at D3; Figure 4D) were observed in the CyPPA group. This group also exhibited a significant increase in the locomotor pattern alteration at D3 (F(6, 42) = 4.254; *p* = 0.412; Figure 4E). These observations indicate that administration of the SK channel agonist CyPPA induces mirroring effects compared to those produced by the SK antagonists. These effects result in an increased severity of vestibular syndrome.

### 2.3. Comparative Study of the Effects of Apamin and Acetyl-DL-Leucine

**Figure 5:**
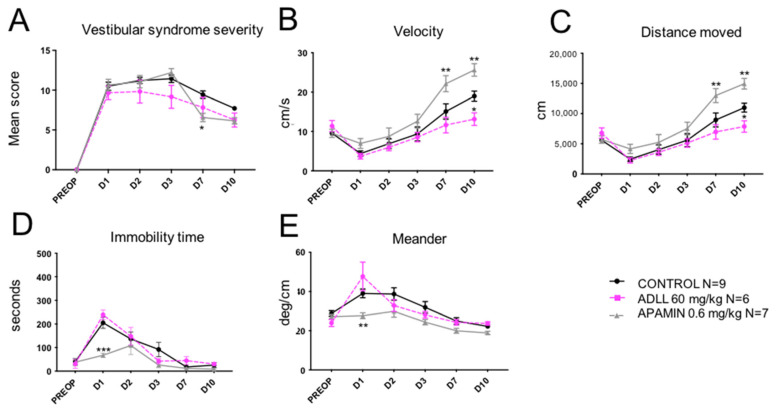
Comparative effects of apamin (0.6 mg/kg) and acetyl-D-L-leucine (60 mg/kg) treatments on vestibular syndrome severity. (**A**), Subjective assessment of the vestibular syndrome severity over time. (**B**), Measurement of the animal’s displacement speed (velocity; in cm/s) at the various time points considered. (**C**), Assessment of the total distance traveled in the open field (in cm) during the recording session. (**D**), Immobility time (in s) of the animals during the videotracking recording phase. (**E**), Locomotor pattern of the animal (meander; °/cm). Statistically significant differences (* *p* < 0.05; ** *p* < 0.01; *** *p* < 0.001) compared to the control group, two-way ANOVA.

Comparative study of the antivertigo effects produced by the ip administration of apamin (0.6 mg/kg) or acetyl-DL-leucine (ADLL; 60 mg/kg) was performed. No statistically significant reduction of the vestibular syndrome severity was evoked by ADLL compared to control or apamin. A significant reduction of the vestibular syndrome severity was observed later at D7 (F(6, 42) = 2.518; *p* = 0.0147; Figure 5A) in the apamin group compared to control or ADLL groups. ADLL administration displayed the opposite effect to apamin from D7, on velocity (Figure 5B) and distance moved (Figure 5C), with significant reduction of both parameters at D10 (F(6, 42) = 4.011; *p* = 0.265 for velocity; F(6, 42) = 5.135; *p* = 0.0445 for distance moved) compared to control groups. ADLL administration also displayed the opposite effect compared to apamin on immobility time (Figure 5D) and altered pattern of locomotion (Figure 5E), as demonstrated by the lack of significant reduction of the immobility time at D1 and the increase of altered locomotion pattern at D1 observed for ADLL. These observations indicated a superior effect of apamin on ADLL in reducing the considered biomarkers of the vestibular syndrome.

## 3. Discussion

### 3.1. Vestibular Syndrome and Electrophysiological Asymmetry in the Vestibular Nuclei

Sudden, unilateral, and complete loss of peripheral vestibular sensory information in humans and animals induces a characteristic vestibular syndrome composed of posturo-locomotor, oculomotor, and vegetative symptoms [15,16]. These deficits are fully or partially compensated over time through a process of neuroplasticity called ‘central vestibular compensation’ [17]. This vestibular syndrome is the result of electrophysiological asymmetry between homologous vestibular nuclei (VN): low spontaneous electrical activity on the deafferented side and high on the intact side [18,19,20]. Data in the literature attest that the reestablishment of the electrophysiological balance between opposing VNs is the key and essential parameter of the post-lesional recovery of postural, locomotor, and gaze stabilization functions [21]. The priority for the deafferented vestibular environment is to restore a level of homeostatic excitability essential for functional restoration. For this purpose, neuronal excitability modulators play a key role. We have recently demonstrated that excitability markers of the deafferented vestibular environment (GABAa receptors and KCC2 cotransporters) reconfigure themselves so that GABA becomes depolarizing and facilitates functional restoration [22]. We also demonstrated that a modification of the post-lesional vestibular environment extends to other modulators of neuronal excitability such as SK channels [13].

### 3.2. Presence of SK Channels in the Vestibular Nuclei and Upregulation of Their Expression Following UVN

The presence of different subtypes of ionic channels sensitive to apamin in the mammal VNs has been proposed on the basis of pharmacological tests [23,24,25]. Our team recently confirmed this at cellular level, demonstrating the presence and distribution of small conductance calcium-activated potassium channels SK1, SK2, and SK3 subunits in the VN complex and associated structures, such as cerebellum and inferior olive, through an autoradiographic approach using radiolabeled apamin [13]. This study also revealed reactive upregulation of SK channels following unilateral vestibular neurectomy (UVN) in ipsi and contralateral VNs at both acute (D7 post-UVN: asymmetric positive regulation) and semi-compensated (D21 post-UVN: symmetrical upregulation) stages of vestibular compensation.

### 3.3. Conservation of the Antivertigo Effect of Apamin across Species

In the present study, we retrieved in vestibulo-injured rats the antivertigo (AV) effect of apamin previously demonstrated in vestibulo-injured cats [13]. We used behavioral assessment parameters widely acknowledged as good indicators of posturo-locomotor deficits in rodents [14,26]. However, the dose of 0.3 mg/kg which caused significant reductions in posturo-locomotor and vestibulo-ocular deficits in the UVN cat model only caused a tendency to improve the various monitored parameters in the UVN rat model, without reaching statistically significant difference vs. sham administration. By contrast, simply doubling the administered apamin dosage was sufficient to achieve significant AV effect vs. control. Doubling the dosage required to produce significant AV effects is not sufficient by itself to be indicative of a real interspecies sensitivity difference. However, it should be noted that the reduction of the posturo-locomotor effects observed in the vestibulo-injured cats with apamin was more pronounced than that observed in rat. Thus, even if the results of the present study initially confirm a conservation of the apamin AV effect between the two species, a difference in sensitivity to apamin cannot be excluded. To what extent this difference may be due to differential expression of SK subunits in cat and rat VN will need to be further investigated.

### 3.4. Tendency of SK Channel Antagonists to Mimic the Antivertigo Effect of Apamin

Apamin is a prototypic ‘blocker’ of SK channels, in fact acting through an allosteric mechanism that physically obstructs the outer channel pore. This action is independent of gating. Apamin is therefore not considered to be a negative modulator [7,27]. Conversely, NS8593, a non-charged molecule with a very different structure from apamin, defined this concept. NS8593 inhibits cloned human and rat SK2 isoforms with equal potency, while it is inactive on SK3. It causes a reduction in the apparent Ca^2+^ sensitivity for channel activation [28]. In the present study, we administrated the compound at 30 mg/kg, a dose previously demonstrated to induce burst firing in mouse dopamine neurons in vivo [29]. At this specific concentration, NS8593 displayed a trend to alleviate all vestibular symptoms considered, although never reaching significant difference vs. control. It can be anticipated that a higher concentration of NS8593 may produce superior and significant antivertigo effect. The difference in both the structure and mechanism of action of NS8593 vs. apamin, especially its lower affinity for the SK2 subunit [30], may support the observed discrepancy between NS8593 and apamin in alleviating the vestibular syndrome evoked in UVN rats.

AG525E1 is a chiral bis-tertiary amine that also differs from the apamin peptide. This compound was initially developed from chemical modulation of laudanosine, with the aim of studying the physiological roles of SK channels in the central nervous system in vivo. AG525E1 has an affinity for SK channels (*K*_i_ = 293 nM) approximately 100-fold higher than the tertiary compound laudanosine (*K*_i_ ∼ 30 μM) and similar to the charged compound dequalinium (*K*_i_ = 221 nM). AG525E1 equipotently blocks SK1, SK2, and SK3 currents in transfected cell lines. Because of its basic and lipophilic properties, it can reach central SK targets [31]. The specific structure and mechanism of action of AG525E1 may explain the observed discrepancy of its AV effect vs. apamin in the rat model of APV. As for NS8593, the tendency of AG525E1 to reduce the vestibular syndrome over the days of its administration supports the idea that the AV effect exerted by the SK channel antagonists operates through the inhibition of the SK2 channel function. However, the non-statistically significant effect may be dependent on its lower affinity for the SK2 subunit compared to apamin. In addition to the question of the affinity of the compounds for the SK subunits, other parameters such as ability to cross the BBB, production of metabolites, related subcellular cascades…) have to be taken into account to decipher the heterogeneity of the behavioral responses.

### 3.5. Mirror Effect of SK2 Selective Activator CyPPA

An interesting observation is the exacerbating effect of cyclohexyl-[2-(3,5-dimethyl-pyrazol-1-yl) -6-methyl-pyrimidin-4-yl] -amine (CyPPA) on the vestibular syndrome observed in vestibulo-injured rats. This effect occurs for certain parameters and at certain time points, upon administration of CyPPA. Conversely to the above-mentioned compounds which show little selectivity between the SK2 family members, CyPPA and its more potent congener NS13001 have fundamentally different structures and also changed selectivity profiles. Both compounds selectively activate SK2.3 and SK2.2 but are completely inactive on SK2.1 and SK3.1 [32,33]. The observed exacerbating effect on the vestibular syndrome is particularly pronounced in the first two days after induction of the lesion, with regard to the assessment of the vertigo syndrome and the mean velocity, and continues over three days for the distance moved, time of immobility, and alteration of the locomotor pattern. It can be anticipated that the selective activation of the SK2.3 and SK2.2 subunits induces a decrease in the spontaneous firing rate of neurons carrying these subunits. This effect may occur through increasing the duration of the apamin-sensitive afterhyperpolarization, and inducing an activity-dependent inhibition of current-evoked action potentials as previously demonstrated in DA neurons from both mouse and rat midbrain slices [29]. This effect could mirror those observed with apamin and other SK channel antagonists, by accenting the SK-channels-mediated syndrome. Of course, it cannot be totally ruled out that CyPPA may produce the observed effect through another pharmacological target such as TRP channels [34].

### 3.6. Superior AV Effect of Apamin vs. Acetyl-DL-Leucine

The comparative study of the antivertigo (AV) effects produced by the administration of apamin or acetyl-DL-leucine (ADLL) in vestibulo-injured rats allows anticipation of the potential of apamin to alleviate the vertigo symptoms compared to the French reference AV compound [35]. Apart from a statistically significant difference in the effect of ADLL vs. control and apamin visible at D3 in the subjective evaluation of vertigo syndrome, the AV effect of apamin is always superior to that of ADLL, regardless of the criteria and time point considered. On this basis, it can thus be anticipated that a significant AV effect of apamin can be obtained in patients suffering from APV.

### 3.7. Antivertigo Effect of SK Antagonists and Control of Neuronal Excitability

The role of SK channels in controlling the rhythmicity of neural action potentials is now well established. It is known that their activation, or increase in membrane expression, induces a reduction in neuronal discharge frequency, while their pharmacological blockade induces the opposite effect: an increase in neuronal firing [10,11,12]. This effect has been confirmed on type B neurons in the medial VN using both in vitro brain stem slice and in vivo electrophysiological recordings [24,25,36]. On the basis of these observations, it is very likely that the antivertigo effect of apamin results mainly from its action of stimulating neuronal excitability. In particular, apamin by blocking SK channels could accelerate the recovery of electrophysiological homeostasis between opposite VNs, thus decreasing the symptoms of acute vertigo attacks and accelerating vestibular compensation.

### 3.8. Other Mechanisms of Action for the Antivertigo Effect of SK Antagonist

Although there is much evidence to support an AV effect of SK channel antagonists occurring through the modulation of VN neuron excitability, it cannot be excluded that the AV effect of apamin may also be exerted through other actions.

The animal model of unilateral vestibular lesion is also a neuroinflammatory reaction model since it induces astroglial [37,38,39,40,41] and microglial [22,41,42,43] reactions, as well as the expression of inflammatory factors (TNF-a and NF-kB) in deafferent VNs [44]. Apamin could display, through an action on the SK channels carried by glial cells (astrocytes and microglia) [45,46], a modulating effect on the central inflammation which occurs within the deafferented VNs after UVN. Apamin could thus slow down or even prevent chronic inflammation consecutive to vestibular lesion. There is a growing interest in developing drugs to target microglia and thereby control neuroinflammatory processes, and apamin is a promising candidate. It was recently shown that apamin significantly inhibits proinflammatory cytokine production and microglial cell activation [47].

The AV action of apamin may also take place through the involvement of serotonin. By blocking the SK channels carried by raphe serotonin neurons projecting directly to the VN [48], apamin could specifically enhance the excitability of brainstem vestibular neurons, as previously demonstrated in a microiontophoretic study in the rat [49,50]. In this same line of thought, the effects of dorsal raphe electrical stimulation exert a generalized excitatory influence by serotoninergic fibers on LVN neurons [51]. It could also have an anxiolytic effect which would be beneficial for the central compensation [52,53]. In fact, apamin increases the activity of serotonergic neurons, inducing an increase in its release at the level of the amygdala, the main nervous center regulating emotions [54].

Through a stimulating action on the release of dopamine, apamin could also induce a normalization of excitability, an improvement in motor control, as well as a motivational effect. By an action on the SK channels expressed by dopaminergic neurons which project directly onto the VN, apamin could, like serotonin, normalize the excitability of the VN neurons, through the dopaminergic receptors they express [55]. It is of interest to mention that regulation of SK channels is also observed in substantia nigra in a rodent model of Parkinson’s disease, and that treatment with apamin improves motor deficits by intensifying the excitability of dopaminergic neurons, resulting in an increase in dopamine secretion [56,57,58,59]. Substantia nigra is the main cerebral source of dopaminergic neurons involved in the regulation of motricity, the progressive degeneration of which is one of the main causes of Parkinson’s disease. A recent review documents the anatomical and functional correlation between Parkinson’s disease and vestibular system dysfunction [59]. There is indeed substantial evidence of Parkinsonian neuropathological changes (Lewy bodies and lipofuscin) in the VN complex [60,61]. The common denominator of these two pathologies is postural instability as the main symptom. Finally, clinical trials in Parkinson’s patients show that bee venom does not induce toxicity and slightly improves motor scores [62]. The administration of the venom through specific acupuncture points appears to be beneficial to idiopathic Parkinson’s patients [63]. Taken together, these data suggest that apamin attenuates locomotor postural deficits resulting from vestibular damage, through an indirect action on the dopaminergic system of the substantia nigra. Finally, through a facilitating action on the release of dopamine in the affectivo-motivational sphere, apamin could also have a motivation booster effect. This aspect is very important in human clinics for functional recovery. It is important to emphasize that it has been observed in vivo and post-mortem that the organization of neurotransmitter systems (dopamine, serotonin, and others) is similar between the rat and man. Thus, these supposed mechanisms of action are probably the same in humans.

### 3.9. Limitations of the Study

In the case of groups AG525E1 and NS8593, only trends of change could be seen, instead of statistical significance. This is likely due to the number of animals used in this study. We can anticipate that a higher number of animals per group would have led to significant results for these specific compounds, as observed for apamin, ADLL, and CyPPA. In the same way, higher doses of AG525E1, NS8593, and CyPPA would have probably induced significant effects. For ethical reasons, we chose to omit vehicle groups, as Kolliphor and HP-ß-CD were never demonstrated to influence the absorption, distribution, or metabolisms of any tested compounds. It has been evidenced that regardless of the dosage or the administration duration (daily injections at 30% up to 91 days for Kolliphor, injections of up to 10g/kg for HP-ß-CD), these compounds never produced overt toxicity in rats [64,65]. Note that we used the following in the present study: doses of 5% Kolliphor and 10% HP-ß-CD in daily administration over only 4 consecutive days.

## 4. Materials and Methods

### 4.1. Animals and Ethical Statements

Experiments were performed on 52 Long Evans male rats 10–12 weeks old (250/300 g) originating from our own breeding, from parents supplied by Charles River (St Germain sur l’Arbresle, France). All experiments were performed in accordance with the National Institutes of Health’s Guide for Care and Use of Laboratory Animals (NIH Publication no. 80-23) revised in 1996 for the UK Animals (Scientific Procedures) Act of 1986 and associated guidelines or the Policy on Ethics approved by the Society for Neuroscience in November 1989 and amended in November 1993 and under the veterinary and National Ethical Committee supervision (French Agriculture Ministry Authorization: B13-055-25). The present study was specifically approved by Neurosciences Ethic Committee N°71 of the French National Committee of animal experimentation. Every attempt was made to minimize both the number and suffering of animals used in this experiment. The animals were housed in a large confined space with 12–12 h diurnal light variations with free access to water and food. They were housed at the LNC UMR 7291 animal facility.

### 4.2. Study Design

**Figure 6:**
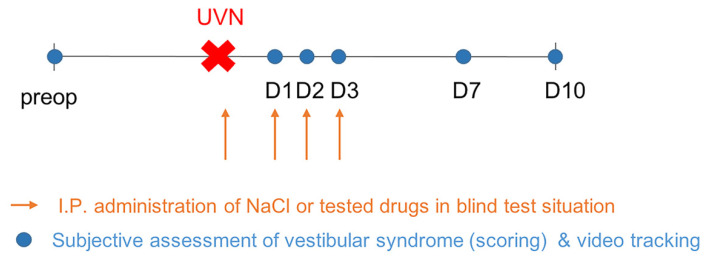
Operating protocol. Upper part: details of the procedure used to evaluate the vestibular syndrome before and after unilateral vestibular neurectomy (red cross). Time of drug administration (orange arrow), behavioral investigation of the posturo-locomotor parameters (blue filled circle).

To determine the impact of SK modulators and acetyl-D-L leucine (ADLL) treatment on the kinetic of vestibular syndrome and vestibular compensation in UVN rats, we administrated the compounds during the acute phase of the vestibular syndrome following the lesion, and we observed the consequences of this treatment at the behavioral level. For each behavioral investigation, acquisitions were performed before surgery (preop) and at different time-points (days) during the post-operative time (D1, D2, D3, D7, and D10) to evaluate the effect of these different treatments on the kinetics of the vestibular syndrome and compensation (Figure 6). Animals were randomly divided into seven groups: UVN + placebo group referred to as *control* group (*n* = 9), lesioned and treated with NaCl 0,9%; two UVN + apamin groups receiving two different doses (0.3 mg/kg; *n* = 6) and (0.6 mg/kg; *n* = 8); UVN+ AG525E1 group (*n* = 8); UVN+ NS8593 group (*n* = 8), UVN + CyPPA (cyclohexyl-[2-(3,5-dimethyl-pyrazol-1-yl) -6-methyl-pyrimidin-4-yl]-amine) group (*n* = 5), and UVN + ADLL group (*n* = 6). The different groups were randomly monitored at each time point. At the behavioral level, we measured the kinetics of the vestibular syndrome and compensation using different evaluations performed at different time points after the lesion.

### 4.3. Unilateral Vestibular Neurectomy

Animals were subjected to a left-side vestibular nerve section under visual control through a dissecting microscope under isoflurane anesthesia 30 min after a subcutaneous injection of buprenorphine (Buprecare^®^; 0.02 mg/kg). The vestibular nerve was sectioned at a post ganglion level as close as possible to the brainstem. Before awakening the animal, a solution of Ringer Lactate (Virbac; 10 mL/kg) was administered subcutaneously in order to alleviate the dehydration resulting from the inability of the animal to drink normally as a consequence of the injury. The success of the surgery (full section of the vestibular nerve) was verified at the histological level by the observation under optical microscopy of the full section of the 8th cranial nerve between Scarpa’s ganglion and brainstem vestibular nuclei (see [66] for details), and confirmed at behavioral level by the expression of characteristic vestibular syndrome composed of an acute phase between D1 and D3 during which the posturo-locomotor deficits reached maximum values, and a compensation phase during which the vestibular deficit was progressively reduced. Animals which did not follow these criteria were not considered in the analysis.

### 4.4. Drugs Administration

The pharmacological treatments were administrated once a day by intraperitoneal (i.p) injections in double blind test conditions, during the acute phase of the vestibular syndrome (30 min following the surgery, and at D1–D3 post UVN). The UVN + placebo group was administrated with NaCl 0.9%. The SK modulators and acetyl-D-L leucine (Tanganil) were administrated at different times before each behavioral test to take into account the molecules’ half-time, and at the following concentrations: apamin (0.3 and 0.6 mg/kg dissolved in NaCl 0.9%, injection 30 min before test, Genepep SA, Montpellier, France), AG525E1 (10 mg/kg, dissolved in NaCl, injection 15 min before test, University of Liege, Liege, Belgium), NS8593 (30 mg/kg diluted in 10% (2-hydroxypropyl)-β-cyclodextrin (HP-β-CD); injection 5 min before test, Pharmaceutical Chemistry Lab, University of Liege, Liege, Belgium), CYPPA (15 mg/kg, diluted in 5% solution of Kolliphor RH40 in saline; injection 10 min before test, Alomone Labs Ltd., Jerusalem, Israel), ADLL (60 mg/kg, injection 15 min before test, gift from Otorhinolaryngology and Head Neck Surgery Department, Conception University Hospital, Marseille, France). Systemic administration of the selected drugs was chosen as the compounds were found to cross the blood-brain barrier [29,31,33,67]. The animals were allocated to different subgroups, allowing comparison between vehicle-lesioned and drug-lesioned rats. For each subgroup, we determined through adapted behavioral tests the effects of the drug treatments on the acute phase of the vestibular syndrome and the recovery of posturo-locomotor functions.

### 4.5. Behavioral Investigations

#### 4.5.1. Qualitative Evaluation of the Vestibular Syndrome

We evaluated the vestibular syndrome intensity and kinetic by using a cumulative qualitative scale listing stereotypical postural and locomotor deficits evoked following UVN adapted from [41]. Each of the following symptoms was scored on the qualitative scale. *Tail hanging behavior*: Animals were picked up from the ground at the base of the tail and body rotation was scored from 0 (no rotation) to 3 (several rotations of 360°). *Rearing*: the ability of the rat to rear was scored from 0 (rearing is observed) to 1 (rearing is absent). *Grooming*: the ability of the rat to groom correctly was scored as follows: 0 (correct grooming of full body) 1 (grooming of the face, belly, and flanks but not the base of the tail), 2 (grooming of the face and belly), 3 (grooming of the face), 4 (inability of the animal to groom itself). *Displacement*: quality of the displacement of the rat was scored from 0 (displacement of the rat with no visible deficit) to 4 (inability of the animal to move forward or presence of retropulsion). *Head tilt* was scored by estimating the angle between the jaw plane and the horizontal with 0 (absence of head tilt) to 3 (for a 90° angle). *Barrel rolling* was scored as follows: 0 (absence of barrel rolling), 1 (barrel rolling evoked by an acceleration in the vertical axis of the rat in our hand), 2 (barrel rolling evoked by handling the animal), 3 (spontaneous barrel rolling). *Circling* was scored as follows: 0 (absence of circling behavior), 1 (rare episodes of circling behavior evoked by handling of the animal), 2 (presence of circling behavior). *Bobbing* is assimilated to cephalic nystagmus and was scored from 0 (absence of bobbing) to 1 (presence of bobbing). Circling was scored as follows: 0 (absence of circling behavior), 1 (rare episodes of circling behavior evoked by handling of the animal), 2 (presence of circling behavior). Circling refers to rapid rotational behavior that is observed in different disease models. This phenotype is common to rodent models of various pathologies with cerebral asymmetry (Parkinson’s disease, schizophrenia, depression, or anxiety). Circling may be a result of striatal electrophysiological imbalances resulting from excitability asymmetries observed in the VN after unilateral vestibular loss (see [66]).

#### 4.5.2. Quantitative Evaluation of the Vestibular Syndrome

Animals were individually placed in an open field (80 cm length × 80 cm width × 40 cm height). Their behavior was recorded for 10 min using a digital camera and analyzed with EthoVision™ XT 14 software (Noldus). The surfaces of the open field were cleaned thoroughly between trials. To minimize stress, the room was lit as dimly as possible, while allowing us to clearly discern the rats. At the beginning of the session, the rat was placed on the right side of the field, head facing the wall. A first acquisition was performed the day before the lesion, serving as a reference value, and then acquisitions were performed at days (D) 1, 2, 3, 7, 10 post-lesion. We used the dynamic subtraction method. EthoVision™ automatically detected the following body points throughout the recordings: nose, body center, and tail base of each animal. We used 3 analysis profiles for 23 variables selected for analysis. We used the total distance moved (cm), the mean velocity (cm/s), the meander, and the immobility time (s) to assess the locomotor activity of the rat. For more details see [16].

#### 4.5.3. Data Treatment and Statistical Analysis

Statistical analyses were evaluated by one-way repeated measures ANOVA followed by a simple contrast to compare the postoperative time with the preoperative time for each group (JASP). The number of animals per group was anticipated using statistical tests (https://biostatgv.sentiweb.fr/?module=etudes/sujets#, accessed on 15 March 2018) with expected effect size 0.6, power 0.85; *p* values: 0.05. Differences between the Sham and NVU group were evaluated by two-way repeated measures ANOVA. If significant effects were found, Dunnett multiple comparisons test was performed (GraphPad, Prism). Results were considered significant at *p* < 0.05. In the Results section, data are presented as statistical value, degrees of freedom, and exact *p* value.

## 5. Conclusions

Altogether, these data confirm the significant antivertigo benefit of apamin in vestibulo-injured rats, with improvement of locomotion and mobility shortly (one day) after the insult, and reduction of syndrome intensity and improvement of both velocity and distance traveled after a week. It can be anticipated that the antivertigo effect of apamin results from a direct action on the normalization of neuronal excitability within the VNs, considered the key parameter of vestibular compensation. Its antivertigo effect could also result from indirect actions on other neural networks, whose modulation would be beneficial for the compensation.

## Data Availability

Data are contained within the article.
